# Detection of a gammaretrovirus, XMRV, in the human population: Open questions and implications for xenotransplantation

**DOI:** 10.1186/1742-4690-7-16

**Published:** 2010-03-10

**Authors:** Joachim Denner

**Affiliations:** 1 Retrovirus induced immunosuppression, Robert Koch Institute, Nordufer 20, D-13353 Berlin, Germany

## Abstract

XMRV (**x**enotropic **m**urine leukaemia virus-**r**elated **v**irus) is a gammaretrovirus that has been detected in human patients with prostate carcinoma, chronic fatigue syndrome (CFS) and also in a small percentage of clinically healthy individuals. It is not yet clear whether the distribution of this virus is primarily limited to the USA or whether it is causally associated with human disease. If future investigations confirm a broad distribution of XMRV and its association with disease, this would have an impact on xenotransplantation of porcine tissues and organs. Xenotransplantation is currently being developed to compensate for the increasing shortage of human material for the treatment of tissue and organ failure but could result in the transmission of porcine pathogens. Maintenance of pathogen-free donor animals will dramatically reduce this risk, but some of the porcine endogenous retroviruses (PERVs) found in the genome of all pigs, can produce infectious virus and infect cultured human cells. PERVs are closely related to XMRV so it is critical to develop tests that discriminate between them. Since recombination can occur between viruses, and recombinants can exhibit synergism, recipients should be tested for XMRV before xenotransplantation.

## Questions concerning XMRV detection

XMRV was first detected in prostate carcinomas of patients who were homozygous for a mutation in the gene for the antiviral enzyme, ribonuclease L (RNase L) [1]. Men with two copies of the homozygous mutation R462Q (QQ) were found to have twice the risk of prostate cancer as males with the non-mutated allele. Integrated XMRV was detected in 8 of 20 of these patients (40%) using a DNA microarray and RT-PCR analysis. In heterozygous patients and patients without the mutation, XMRV was found only in 1.5% of prostate tumours. The sequence of the virus is closely related (more than 93% DNA sequence identity) to other xenotropic murine retroviruses. Xenotropic viruses infect only cells from other species. Interestingly, low levels of XMRV protein expression were detected in a small number of stromal cells, but not in the tumour cells themselves. *In vitro *tests have revealed that the virus productively infects human cells and that its replication is susceptible to IFN-β treatment [2]. Another study identified XMRV proviral DNA in 6% of the prostate tumours analysed by real time PCR and viral protein was detected in 23% of 334 prostate tumours using antisera against a panel of murine retroviruses including XMRV [3]. In that study, infection was associated with high-grade tumours, but did not correlate with the RNase L QQ variant. In contrast to previous reports [1], XMRV proteins were found to be expressed primarily in tumour cells [3]. Unfortunately screening for XMRV specific antibodies was not performed, although it is generally agreed that detection of antibodies is a common and reliable diagnostic method to detect low level infections with retroviruses including HIV-1. In cases of low proviral load, antibody detection can indicate infection in the absence of positive PCR results [4].

In Europe, XMRV appears to be less common than in the USA. No XMRV was found in 139 Irish prostate cancer patients with the RNase L mutation [5]. In a German study, XMRV-specific sequences were detected in only in 1 of 105 tissue samples from non-familial prostate cancer and in 1 of 70 tissue samples from men without prostate cancer [6]. The two positive samples were not correlated with homozygosity for the R462Q mutation. In a larger study, Hohn et al. [6] failed to find any XMRV-specific sequences in the DNA and RNA from tumours of 589 German prostate cancer patients, even though 12.9% of them were shown to be of the QQ genotype [7]. Most importantly, we did not find antibodies when sera from 146 patients from this cohort were tested [7].

A recent study in the USA identified XMRV-specific proviral DNA in PBMCs from patients suffering from chronic fatigue syndrome (CFS) [8]. CSF is characterized by debilitating fatigue, chronic inflammation and other abnormalities of the immune system such as a deficiency in natural killer cell activity. In 68 of 101 CFS patients (67%), but also in 8 of 218 (3.7%) healthy controls, XMRV could be detected by PCR [8]. This 3.7% incidence in healthy controls suggests that several million Americans may be infected. Laboratory tests revealed that virus from patient-derived sera could infect cultured human cells. The authors detected antiviral antibodies in 9 out of 18 patients using a test based on an envelope protein from spleen focus forming virus which is closely related to XMRV. In contrast, two other studies found no XMRV in clinically well-characterized European CFS patients [9,10]. In one of these studies, PCR analysis revealed no XMRV DNA in 299 samples, although some serum samples showed XMRV neutralising activity. Only one of these was from a CFS patient [9]. In the other study, neither XMRV nor murine leukemia virus sequences were detected. The authors came to the conclusion the XMRV is not a contributory factor in the pathogenesis of CSF [10].

In light of these results, the finding of XMRV in prostate tumours and CFS patients in the USA has to be treated with great caution. In the past, numerous retroviruses have been reported in human tissues and cultured cells (for review see [11]). Some of these have later been shown to be contaminating animal viruses with unknown origin and function. Only HIV-1 and HIV-2, both of which induce acquired immunodeficiency syndromes (AIDS), and the human T-lymphotropic viruses, HTLV-1 and HTLV-2, which induce adult T-cell leukaemia and HTLV-associated myelopathy/tropical spastic paraperesis, have been proven to be linked to human diseases. Highly sensitive methods such as PCR allow detection of minimal traces of retroviruses but also detect sample contamination. Indeed, contaminating retroviruses, mainly gammaretroviruses, have been found in numerous human cell lines (for literature see the latest report concerning this topic, [12]).

Trans-species transmission of retroviruses has occurred frequently during evolution (for review see [13]), and HIV-1 is the best-known example. Trans-species transmission has also been reported for gammaretroviruses closely related to XMRV, for example the Koala retrovirus [14]. Important questions remain to be answered: How and when did this murine xenotropic virus infect humans? Is it a direct infection from rodents to susceptible humans or is the virus spreading through human populations resulting in high virus load in (immunosuppressed) tumours and CFS patients? Is a third species transmitting the virus from mice to humans? As mentioned above, 3.7% of healthy controls from one study in the USA were virus positive [3]. Why is the virus common in the USA but rare in Europe? Are there specific populations of rodents in the USA releasing this virus? Why is disease associated with XMRV in the USA but not in Europe? Is XMRV a passenger virus replicating in immuno-compromised individuals or does this virus contribute directly to disease progression? The immunosuppressive properties characteristic of all retroviruses [15] could contribute to tumour progression as well as to the symptoms of CSF. If XMRV is indeed widely distributed in the human population and associated with tumours and CSF, should blood donors be tested in order to avoid XMRV transmission?

## PERVs, XMRV and possible implications for xenotransplantation

With so many unanswered questions, much work remains to be done. At this early point in XMRV research, areas should be identified in which this virus may cause a serious impact on human health. One of these areas may be the xenotransplantation of porcine tissues and organs to humans. Xenotransplantation is a potential solution for the shortage of allogeneic human organs. Designated pathogen-free breeding and maintenance of pigs can prevent the transmission of most porcine pathogens; however, porcine endogenous retroviruses (PERVs) are integrated in the pig genome and can be released from normal pigs and infect human cells in vitro [16,17]. In the past, highly sensitive methods had been developed to detect PERV infection. A PCR study of more than 200 patients treated with pig material for PERV transmission found no viral DNA [18]. However, three of the patients showed a clear antibody response against the p27Gag of PERV in a Western blot assay, and a small percentage of blood donors (5%) have also been found to react with the p27Gag of PERV [18,19]. Since antibodies against the Env protein of PERV were not found, this reaction was classified as not specific for PERV following the rules applied for HIV-1 diagnostic tests. Is it possible that the antibody response detected was against a related retrovirus such as XMRV?

If XMRV is indeed circulating in the human population, it has important implications for xenotransplantation. A test should be developed to discriminate between PERV and XMRV, and the potential for recombination between the two viruses should be investigated. Recombination between the human tropic PERV-A and the ecotropic PERV-C has been described in normal pigs and in melanoma-bearing animals, and recombinant PERV-A/C was characterized by high replication titers [20-22]. Whether XMRV and PERV recombine remains unclear, however co-packaging [23] and pseudotyping [24] between PERV and murine retroviruses have been described. Although the sequence identity between PERV and XMRV is only approximately 53%, there are regions with higher homology that would allow recombination.

This raises new questions: Should the xenotransplant recipients be pre-screened for XMRV to avoid recombination? What measures can be taken when XMRV infection is detected in such a screen? Before dealing with these specific details, it is necessary to address the important broad questions concerning the distribution of XMRV and its impact on human health.

## Competing interests

The author declares that he has no competing interests.

## Added in proof

In a recent case-control study van Kuppeveld et al. [25] detected no XMRV sequences in any of the Dutch patients with CFS or controls.
